# Chronic kidney disease and survival following indirect mitral annuloplasty for functional mitral regurgitation

**DOI:** 10.3389/fcvm.2025.1622875

**Published:** 2025-12-02

**Authors:** Dennis Rottländer, Milad Golabkesh, Hubertus Degen, Dimitrios Barlagiannis, Alev Ögütcü, Martin Saal, Michael Haude

**Affiliations:** 1 Department of Cardiology, Faculty of Health, School of Medicine, University Witten/Herdecke, Witten, Germany; 2 Department of Cardiology, Krankenhaus Porz am Rhein, Cologne, Germany; 3 Department of Cardiology, Rheinland Klinikum Neuss, Neuss, Germany

**Keywords:** transcatheter mitral valve repair, functional mitral valve regurgitation, Carillon, acute kidney injury, chronic kidney disease

## Abstract

**Background:**

Chronic kidney disease is associated with poor prognosis following mitral valve edge-to-edge repair. We aimed to investigate the impact of chronic kidney disease (CKD) on survival in patients with functional mitral regurgitation (FMR) undergoing indirect mitral annuloplasty using the Carillon Mitral Contour System.

**Methods:**

In a retrospective analysis, a total of 100 consecutive FMR patients were assigned according to baseline renal function into three groups: estimated Glomerular Filtration Rate (eGFR) > 60 mL/min/1.73 m^2^, eGFR 30–59 mL/min/1.73 m^2^ and eGFR < 30 mL/min/1.73 m^2^. At 3- and 12-months follow-up after indirect mitral annuloplasty survival, NYHA classification and transthoracic echocardiography were evaluated.

**Results:**

30 patients revealed a baseline eGFR > 60 mL/min/1.73 m^2^ (30%), 51 an eGFR 30–59 mL/min/1.73 m^2^ (51%) and 19 patients an eGFR < 30 mL/min/1.73 m^2^ (19%). 1-year mortality was significantly higher in eGFR 30–59 mL/min/1.73 m^2^ and eGFR < 30 mL/min/1.73 m^2^ compared to a preserved renal function of eGFR > 60 mL/min/1.73 m^2^ (Log Rank test *P* value: 0.036). FMR patients with postprocedural acute kidney injury (AKI), defined as an increase in serum creatinine levels ≥0.3 mg/dL within 48 h or ≥1.5 times baseline within 7 days post procedure, showed significantly increased mortality after indirect annuloplasty (Log Rank test *P* Value: 0.002). Carillon device implantation resulted in FMR reduction and improved NYHA classification at 3- and 12-months follow-up regardless of CKD.

**Conclusion:**

CKD in patients undergoing indirect mitral annuloplasty seems to be a negative predictor of outcome in FMR patients. However, Carillon device implantation improved NYHA and FMR classification regardless of renal function.

## 1 Introduction

Indirect mitral annuloplasty using the Carillon Mitral Contour System represents a treatment option for patients with functional mitral regurgitation (FMR) and heart failure. The Carillon Mitral Contour System is a CE-marked, percutaneous device approved for FMR treatment and is fully reimbursable in Germany. Besides reducing mitral regurgitant volume and promoting left ventricular (LV) remodeling, functional patient outcomes, such as improvements in NYHA class, have been observed (1–3). Moreover, indirect mitral annuloplasty is associated with left atrial remodeling in FMR (4).

In heart failure patients, chronic kidney disease (CKD) is known to be associated with reduced survival (5–7). Transcatheter edge-to-edge repair (TEER) has been shown to improve clinical outcomes in heart failure patients with FMR with refractory symptoms despite guideline-directed medical therapy (GDMT) (8). CKD is associated with poorer outcomes in heart failure patients undergoing TEER for FMR (9, 10). Acute kidney injury (AKI) has been identified as an important predictor of survival following mitral TEER (11). Furthermore, improved renal function after mitral edge-to-edge repair is associated with a reduction in NYHA class and decreased hospitalization rate for heart failure (12).

The effects of CKD in patients undergoing indirect mitral annuloplasty are unknown. This study aimed to evaluate the impact of CKD and AKI on outcome following Carillon device implantation in patients with FMR using a large-scale single center registry from Germany.

## 2 Materials and methods

### 2.1 Patient cohort

The study was performed in compliance with the Helsinki declaration and according to good clinical practice. Individual written consent was provided by all enrolled patients. The ethical committee of the University of Witten-Herdecke (approval number: S-129/2024) approved the conduction of this study. We performed a retrospective single-center analysis of 100 consecutive patients diagnosed with FMR undergoing indirect mitral valve annuloplasty using the Carillon Mitral Contour System (Cardiac Dimensions, Kirkland, WA, USA) based on a local multidisciplinary heart team decision. All patients were on guideline-directed stable heart failure medication (13, 14). Exclusion criteria included patients with degenerative mitral regurgitation not suitable for Carillon implantation. Patients were divided into three groups according to CKD stages: group 1: eGFR > 60 mL/min/1.73 m^2^ (G1-2); group 2: eGFR 30–59 mL/min/1.73 m^2^ (G3) and group 3: eGFR < 30 mL/min/1.73 m^2^ (G4-5). Of note, only one patient had a baseline eGFR > 90 mL/min/1.73 m^2^ (G1); therefore, a separate group for these patients was not established. All patients reported symptomatic (≥NYHA II) heart failure with at least moderate functional mitral regurgitation (FMR ≥ 2+) (15). We further stratified our patient cohort into two subgroups based on postprocedural acute kidney injury (AKI). AKI was classified using the serum creatinine criteria of the 2012 KDIGO guideline (increase ≥0.3 mg/dL within 48 h or ≥1.5 times baseline within 7 days), as urine output measurements were not consistently available (16). All episodes of AKI were transient, with complete recovery of kidney function observed before hospital discharge.

**Figure 1:**
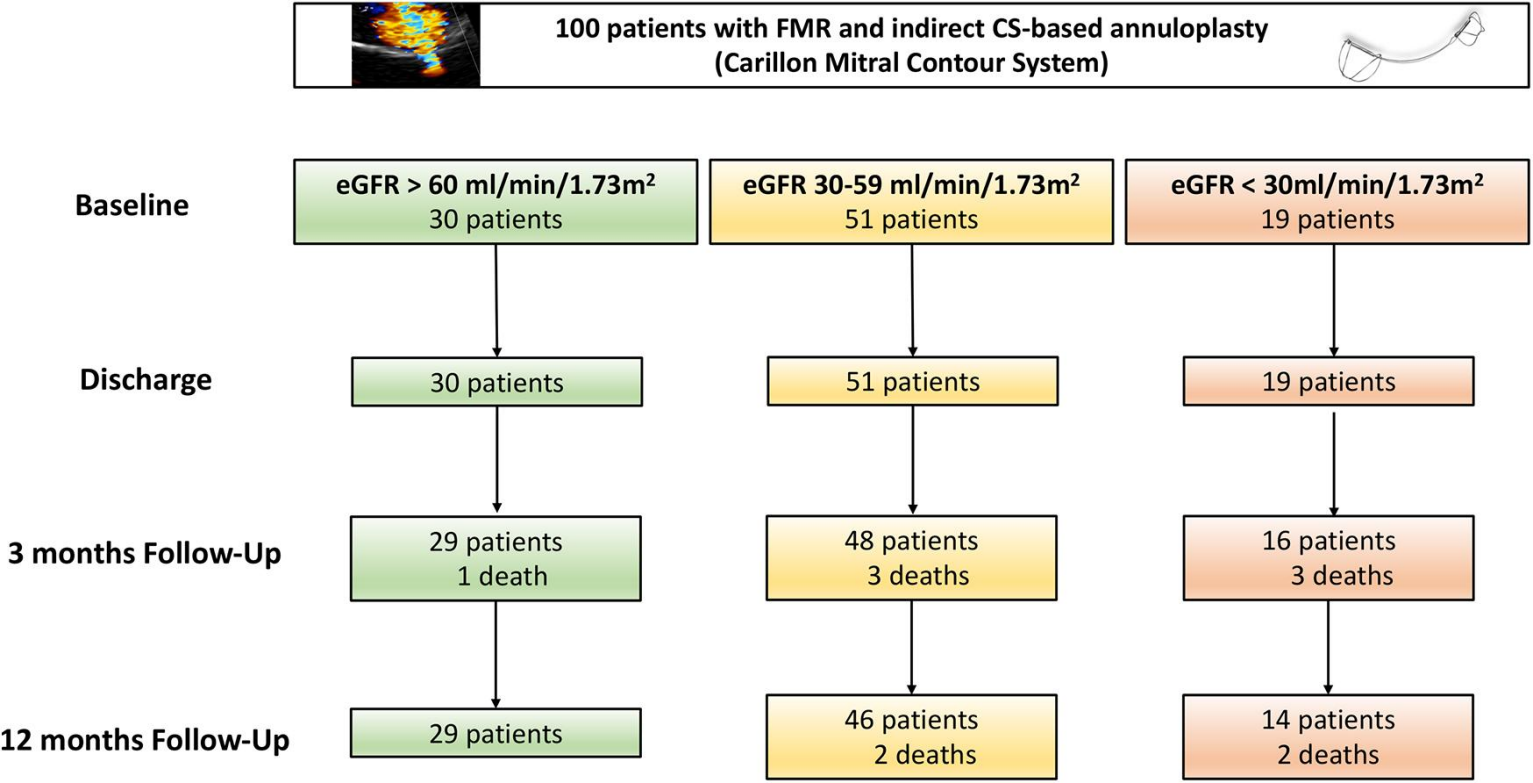
Study flow chart. CS, coronary sinus; FMR, functional mitral regurgitation; eGFR, estimated glomerular filtration rate.

Baseline characteristics as well as 3-month and 12-month clinical follow-ups were obtained for these cohorts. Additionally, transthoracic echocardiography (TTE) follow-ups were conducted to assess and quantify functional mitral regurgitation classification (17). No patients were lost to follow-up during the study. The median follow-up duration was 392 days as all patients had either in-person or structured telephone follow-up at 12 months. However, not all patients underwent TTE at follow-up. Some patients were assessed via structured telephone interviews to evaluate NYHA classification instead. NT-proBNP values were log-transformed using the natural logarithm (ln) prior to all statistical analyses to address non-normal distribution and satisfy model assumptions. Figure 1 shows the study flow diagram.

### 2.2 Carillon mitral contour system

Patients with symptomatic heart failure (≥NYHA II) and at least moderate FMR (FMR ≥ 2+) were eligible for percutaneous annuloplasty using the coronary sinus (CS) based Carillon Mitral Contour System) as previously reported (15).

Briefly, the Carillon device consists of two self-expanding nitinol anchors connected by a nitinol curvilinear segment. Deployment is achieved through a 10 French delivery catheter inserted via right transjugular access. Following unsheathing, the distal anchor is positioned and secured in the coronary sinus (CS). Subsequently, tension is applied to the device, inducing an anterior movement of the posterior part of the mitral annulus and the posterior mitral valve leaflet, thereby enhancing coaptation and reducing FMR. The procedure is completed by deploying the proximal anchor near the coronary sinus ostium to maintain the applied tension. To minimize the risk of coronary artery compression by the device, repeat coronary angiography is performed throughout the procedure. Continuous transesophageal echocardiography (TOE) is performed during the entire procedure to monitor and assess mitral valve regurgitation.

### 2.3 Echocardiography

Baseline and follow-up TTE measurements were performed using a Philips iE 33 or EPIQ echocardiography system (Philips, Amsterdam, Netherlands). The echocardiographic parameters for quantitative assessment of the mitral valve [Vena contracta, proximal isovelocity surface area (PISA), effective regurgitant orifice area (EROA), and regurgitant volume (RegVol)] were assessed. The severity of mitral regurgitation was assessed according to a standard classification system (17). Left ventricular ejection fraction (LVEF) was calculated using Simpsons method in 4- and 2-chamber view.

### 2.4 Renal function

All patients underwent renal laboratory assessments at baseline measuring serum creatinine, blood urea nitrogen (BUN), and estimated glomerular filtration rate (eGFR). The eGFR was calculated using the CKD-EPI formula (18). Classification of CKD stage was performed according to eGFR measurements in conjunction with patients' medical records.

### 2.5 Statistical analysis

Statistical analysis was performed using SPSS Statistics 19 software (IBM, Chicago, USA). The Kolmogorov–Smirnov test was applied to assess the normality of variable distributions. For normally distributed data, results are presented as mean ± standard error of the mean (SEM). The Kruskal–Wallis test was applied for discrete variables, while one-way ANOVA with Tukey *post hoc* tests was used to evaluate continuous variables across groups and subgroups. Ordinal data were analyzed using the Mann–Whitney *U*-test. Kaplan–Meier curves were generated to illustrate long-term survival, and statistical significance was assessed using the Log-Rank test. One-year event rates were calculated using the product-limit estimator, with statistical significance defined as a *p*-value <0.05. Multivariable Cox regression analysis for 1-year mortality was conducted for all Carillon patients. All variables were selected based on a preceding univariate Cox regression analysis, with a *p* < 0.1 for inclusion in the multivariable model. To analyze changes in NYHA classification and FMR severity over time, linear mixed-effects modeling was performed. Fixed effects included timepoint (baseline, 3 months, and 12 months), CKD group (eGFR > 60 mL/min/1.73 m^2^, eGFR 30–59 mL/min/1.73 m^2^, eGFR < 30 mL/min/1.73 m^2^), and the interaction between timepoint and CKD group (timepoint × renal function). An interaction analysis was conducted by including a product term between CKD group and AKI status in the Cox regression model to assess potential effect modification.

## 3 Results

From 100 consecutive FMR patients treated with indirect mitral annuloplasty, 30 patients (30%) were classified in the CKD group 1 (eGFR > 60 mL/min/1.73 m^2^), 51 (51%) in the CKD group 2 (eGFR 30–59 mL/min/1.73 m^2^) and 19 patients (19%) in the CKD group 3 (eGFR < 30 mL/min/1.73 m^2^; Figure 1). Patients baseline characteristics including laboratory results and echocardiographic parameters are shown in Table 1. The majority of the patients in all three groups were male (Table 1). Cardiovascular risk factors including arterial hypertension, smoking status, coronary artery disease were similar across the groups. Patients of group 2 and 3 reported significantly higher rates of diabetes (group 1: 13.3%, group 2: 25.5%, group 3: 47.4%; *p* = 0.029) and previous heart surgery (group 1: 6.7%, group 2: 31.4%, group 3: 26.3%; *p* = 0.035).

**Table 1:** Patients characteristics.

Parameters	eGFR > 60 mL/min/1.73 m^2^ *n* = 30		eGFR 30–59 mL/min/1.73 m^2^ *n* = 51		eGFR < 30 mL/min/1.73 m^2^ *n* = 19		*P*-Value
	n	% or Mean ± SEM	*n*	% or Mean ± SEM	*n*	% or Mean ± SEM	
Age	30	79.2 ± 1.8	51	79.7 ± 1.1	19	82.0 ± 2.2	0.550
Male	21	70.0	31	60.8	14	73.7	0.522
Patients history							
Arterial hypertension	27	90.0	49	96.1	17	89.5	0.476
Diabetes mellitus	4	13.3	13	25.5	9	47.4	0.029
Smoker	4	13.3	9	17.7	1	5.3	0.419
Coronary artery disease	16	53.3	34	66.7	15	79.0	0.179
Previous heart surgery	2	6.7	16	31.4	5	26.3	0.035
Atrial fibrillation	22	73.3	41	80.4	15	88.0	0.761
Transthoracic echocardiography							
LVEF (%)	30	42.7 ± 2.5	51	41.8 ± 1.7	19	35.3 ± 3.0	0.114
LVEDD (mm)	30	53.6 ± 1.3	51	55.0 ± 1.1	19	55.3 ± 1.8	0.657
Mitral annulus diameter (cm)	30	4.2 ± 0.1	51	4.3 ± 0.1	19	4.4 ± 0.1	0.613
PASP (mmHg)	30	43.0 ± 2.0	51	45.7 ± 2.0	19	49.4 ± 3.9	0.296
TAPSE (mm)	30	19.8 ± 0.9	51	17.4 ± 0.7	19	15.5 ± 1.0	0.009
Heart failure							
NICM	8	26.7	16	31.4	5	26.0	0.282
ICM	10	33.3	17	33.3	10	53.0	0.296
HFpEF	12	40.0	18	35.3	4	21.0	0.383
HFmrEF	8	26.7	14	27.4	3	15.8	0.590
HFrEF	10	33.3	19	37.3	12	63.2	0.089
FMR grading							
aFMR	12	40.0	18	35.3	4	21.0	0.383
vFMR	18	60.0	33	64.7	15	79.0	0.383
FMR 1+	0	0.0	0	0.0	0	0.0	1.000
FMR 2+	1	3.3	0	0.0	0	0.0	0.314
FMR 3+	15	56.7	16	31.4	6	31.6	0.216
FMR 4+	14	40.0	35	68.6	13	68.4	0.120
Vena contracta (mm)	30	5.5 ± 0.2	51	6.2 ± 0.2	19	6.2 ± 0.3	0.024
EROA (cm^2^)	30	0.2 ± 0.02	51	0.3 ± 0.01	19	0.3 ± 0.02	0.117
PISA (mm)	30	7.2 ± 0.3	51	7.4 ± 0.2	19	6.9 ± 0.3	0.437
Regurgitant volume (mL)	30	31.2 ± 2.1	451	37.1 ± 2.4	19	38.7 ± 4.0	0.182
Dyspnea							
NYHA II	3	10.0	4	7.8	1	5.3	0.840
NYHA III	20	66.7	35	68.6	12	63.1	0.912
NYHA IV	7	23.3	12	23.6	6	31.6	0.768
Laboratory results							
ln NT-proBNP	28	8.2 ± 0.9	50	8.3 ± 0.7	19	8.9 ± 0.7	0.008
Serum creatinine (mg/dL)	30	1.0 ± 0.1	51	1.4 ± 0.1	19	2.5 ± 0.2	<0.001
eGFR (mL/min/1.73 m^2^)	30	73.5 ± 1.9	51	46.7± 1.2	19	23.9 ± 1.1	<0.001
Baseline dialysis	0	0.0	0	0.0	1	5.3	0.118
Hemoglobin (g/dL)	30	12.7 ± 0.4	51	12.9 ± 0.2	19	11.9 ± 0.6	0.168
Baseline medication							
Beta blocker	28	93.3	49	96.1	19	100.0	0.518
ACEI or ARB or ARNI	26	86.7	47	92.2	16	84.2	0.576
MRA	14	46.7	21	42.2	6	31.6	0.586
Diuretic	29	96.7	49	96.1	19	100.0	0.695

SEM, standard error of the mean; EF, ejection fraction; LV, left ventricle; LVEF, left ventricular ejection fraction; LVEDD, left ventricular enddiastolic diameter; PASP, systolic pulmonary artery pressure; TAPSE, tricuspid annular plane systolic excursion; NICM, non-ischemic cardiomyopathy; ICM, ischemic cardiomyopathy; HFpEF, heart failure with preserved ejection fraction; HFmrEF, heart failure with mildly reduced ejection fraction; HFrEF, heart failure with reduced ejection fraction; FMR, functional mitral regurgitation; aFMR, atrial functional mitral regurgitation; vFMR, ventricular functional mitral regurgitation; EROA, effective regurgitant orifice area; PISA, proximal isovelocity surface Area; NT-proBNP, N-terminal pro-B-type natriuretic peptide; ln, natural logarithm; NYHA, New York Heart Association; eGFR, estimated glomerular filtration rate; ACEI, angiotensin-converting enzyme inhibitor; ARB, angiotensin receptor blocker; ARNI, angiotensin receptor neprilysin inhibitor; MRA, mineralocorticoid receptor antagonist.

In our cohort, atrial FMR was present in 34% of patients and ventricular FMR was present in 66% of patients. Atrial FMR was observed in 40% of patients with eGFR ≥ 60 mL/min, 35.3% with eGFR 30–59 mL/min/1.73 m^2^, and 21% with eGFR < 30 mL/min/1.73 m^2^, whereas ventricular FMR was present in 60%, 64.7%, and 79% of patients in the respective groups.

There was no difference in baseline LVEF, left ventricular enddiastolic diameter (LVEDD) and pulmonary artery systolic pressure (PASP), as measured by baseline TTE, while tricuspid annular plane systolic excursion (TAPSE) was significantly lower in group 2 and 3 (group 1: 19.8 ± 0.9 mm, group 2: 17.4 ± 0.7 mm, group 3: 15.5 ± 1.0 mm; *p* = 0.009; Table 1).

Baseline assessment of mitral regurgitation using transthoracic echocardiography (TTE) showed no differences in FMR classification among the groups (see Table 1). Also, NYHA classification was consistent across the groups (Table 1).

NT-proBNP was significantly higher in group 3 at baseline (natural logarithm of NT-proBNP: group 1: 8.2 ± 0.9, group 2: 8.3 ± 0.7, group 3: 8.9 ± 0.7; *p* = 0.008). Renal baseline parameters are displayed in Table 1.

The rate of procedural complications associated with Carillon device implantation remained low. One minor bleeding incident and a single case of coronary sinus perforation occurred, which was successfully managed with balloon occlusion of the coronary sinus. No procedures were discontinued due to unsuccessful implantation.

**Figure 2:**
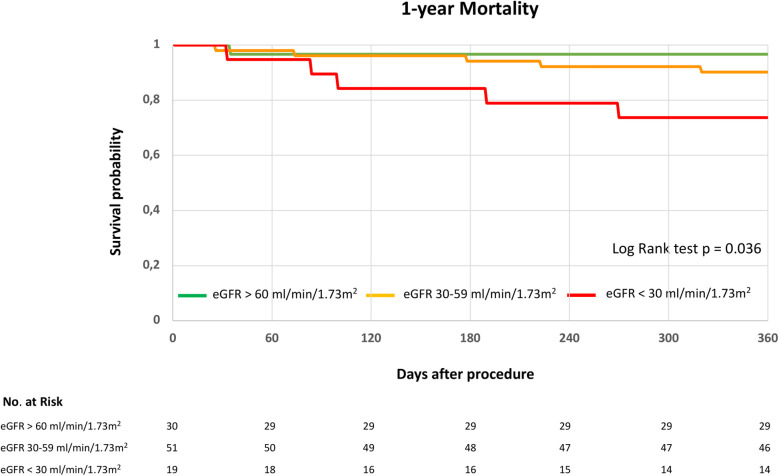
1-year survival following indirect mitral annuloplasty in functional mitral regurgitation according to baseline renal function. Kaplan–Meier curve for 1-year mortality in patients with eGFR > 60 mL/min/1.73 m^2^, eGFR 30–59 mL/min/1.73 m^2^ or eGFR < 30 mL/min/1.73 m^2^. eGFR, estimated glomerular filtration rate. Log rank test was applied to detect statistical differences between the groups (eGFR > 60 mL/min/1.73 m^2^, eGFR 30–59 mL/min/1.73 m^2^ and eGFR < 30 mL/min/1.73 m^2^).

At 30 days, mortality was 0% in the eGFR > 60 mL/min/1.73 m^2^ group (0/30), 2% in the eGFR 30–59 mL/min/1.73 m^2^ group (1/51), and 0% in the eGFR < 30 mL/min/1.73 m^2^ group (0/19). The Kaplan–Meier survival curves illustrate long-term survival stratified by the three eGFR groups (Figure 2). One-year mortality was highest in group 3, followed by group 2, and was lowest in group 3 (log-rank test, *p* = 0.036). At 12-months follow-up, one death was reported in group 1, five deaths in group 2, and five deaths in group 3 (Figure 2).

**Figure 3:**
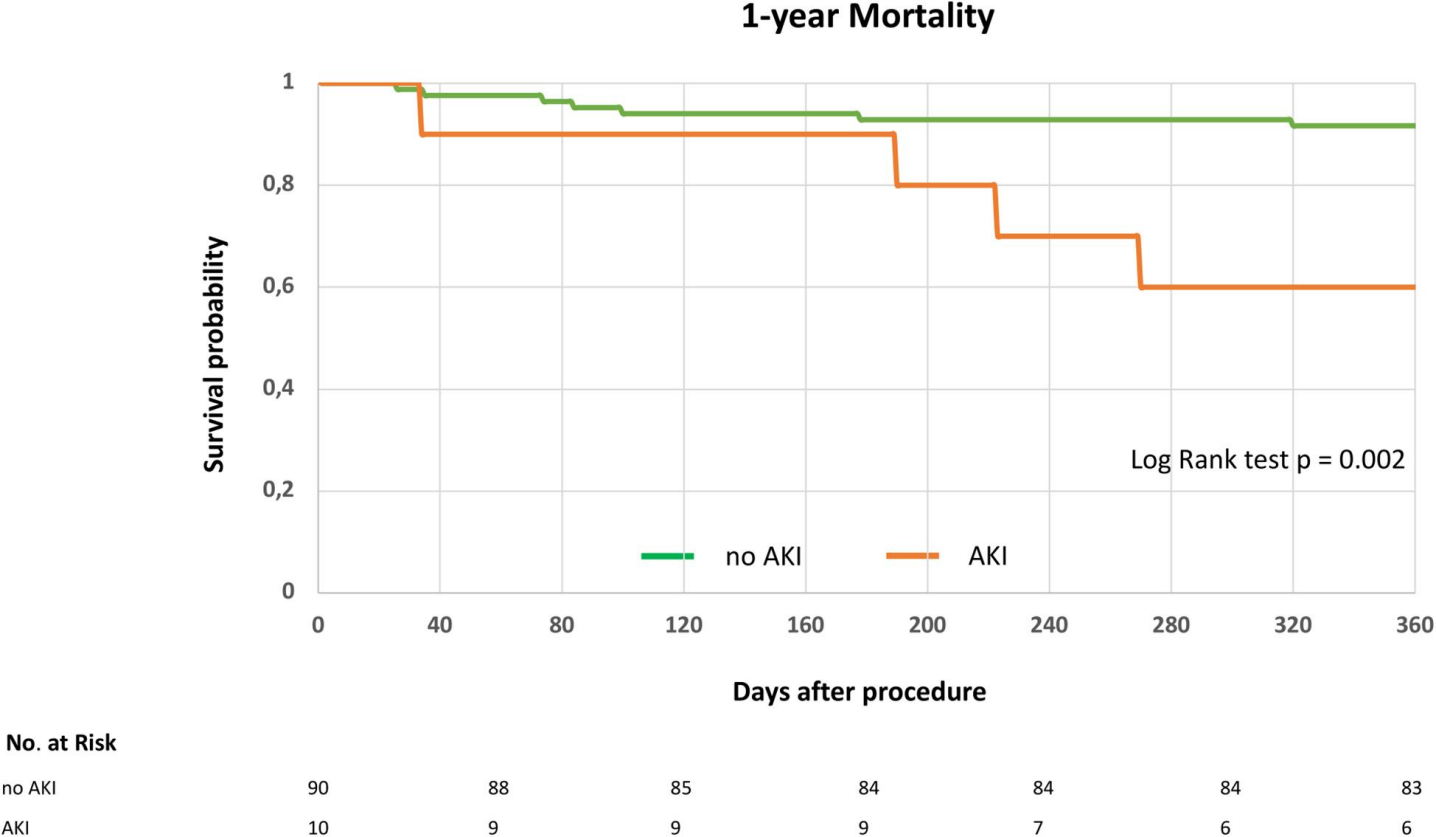
1-year survival following indirect mitral annuloplasty in functional mitral regurgitation according to postprocedural acute kidney injury. Kaplan–Meier curve for 1-year mortality in patients with or without acute kidney injury (AKI). Log rank test was applied to detect statistical differences between the groups (AKI and no AKI).

In our cohort, AKI occurred in 10% of patients (10/100). Baseline characteristics are displayed in Table 2. In patients with AKI, baseline serum creatinine levels were significantly higher, eGFR was significantly lower, and coronary artery disease was more frequently observed compared to the no-AKI group (Table 2). One-year survival was significantly lower in patients who developed postprocedural AKI (AKI: 4 deaths vs. no AKI: 7 deaths; log-rank test, *p* = 0.002; Figure 3). 30-day mortality was 0% (0/10) in the AKI group and 1.1% (1/90) in the no AKI group. No patient required dialysis due to AKI, and only one patient was on chronic dialysis at baseline.

**Table 2:** Patients characteristics of the AKI and no AKI group.

Parameters	No AKI *n* = 90		AKI *n* = 10		*P*-Value
	*n*	% or Mean ± SEM	*n*	% or Mean ± SEM	
Age	90	80.3 ± 0.9	10	83.1 ± 2.8	0.367
Male	59	65.5	7	70.0	0.778
Patients history					
Arterial hypertension	84	93.3	9	90.0	0.695
Diabetes mellitus	23	25.6	3	30.0	0.761
Smoker	14	15.6	0	0.0	0.179
Coronary artery disease	55	61.1	10	100.0	0.014
Previous heart surgery	19	21.1	4	40.0	0.178
Atrial fibrillation	72	80.0	6	60.0	0.148
Transthoracic echocardiography					
LVEF (%)	90	40.5 ± 1.4	10	42.3 ± 4.2	0.681
LVEDD (mm)	90	54.8 ± 0.8	10	52.9 ± 2.4	0.454
PASP (mmHg)	90	45.9 ± 1.5	10	42.3 ± 3.6	0.439
TAPSE (mm)	90	17.8 ± 0.5	10	17.1 ± 0.9	0.647
FMR Grading					
FMR 1+	0	0.0	0	0.0	1.000
FMR 2+	1	1.1	0	0.0	0.738
FMR 3+	35	38.9	2	20.0	0.216
FMR 4+	54	60.0	8	80.0	0.194
Dyspnea					
NYHA II	7	7.8	1	10.0	0.806
NYHA III	59	65.6	8	80.0	0.357
NYHA IV	24	26.7	1	10.0	0.248
Laboratory results					
Serum creatinine (mg/dL)	90	1.4 ± 0.1	10	1.9 ± 0.2	0.020
eGFR (mL/min/1.73 m^2^)	90	55.3 ± 2.2	51	33.2± 5.8	0.001
Baseline dialysis	1	1.1	0	0.0	0.738
Baseline medication					
Beta blocker	86	95.6	10	100.0	0.496
ACEI or ARB or ARNI	80	88.9	9	90.0	0.579
MRA	37	41.1	4	40.0	0.946
Diuretic	87	96.7	10	100.0	0.558

SEM, standard error of the mean; EF, ejection fraction; LV, left ventricle; LVEF, left ventricular ejection fraction; LVEDD, left ventricular enddiastolic diameter; PASP, systolic pulmonary artery pressure; TAPSE, tricuspid annular plane systolic excursion; NYHA, New York Heart Association; eGFR, estimated glomerular filtration rate; ACEI, angiotensin-converting enzyme inhibitor; ARB, angiotensin receptor blocker; ARNI, angiotensin receptor neprilysin inhibitor; MRA, mineralocorticoid receptor antagonist.

At baseline, TTE was available for all of patients (*n* = 100). At 3-month follow-up, TTE was performed in 29/30 (97%) patients with eGFR > 60 mL/min/1.73 m^2^ (1 death), 48/51 (94%) with eGFR 30–59 mL/min/1.73 m^2^ (3 deaths), and 16/19 (84%) with eGFR < 30 mL/min/1.73 m^2^ (3 deaths). At 12 months, TTE was available in 25/30 (83%) patients with eGFR > 60 mL/min/1.73 m^2^ (4 missing), 44/51 (86%) with eGFR 30–59 mL/min/1.73 m^2^ (2 deaths, 2 missing), and 13/19 (68%) with eGFR < 30 mL/min/1.73 m^2^ (2 deaths, 1 missing).

**Figure 4:**
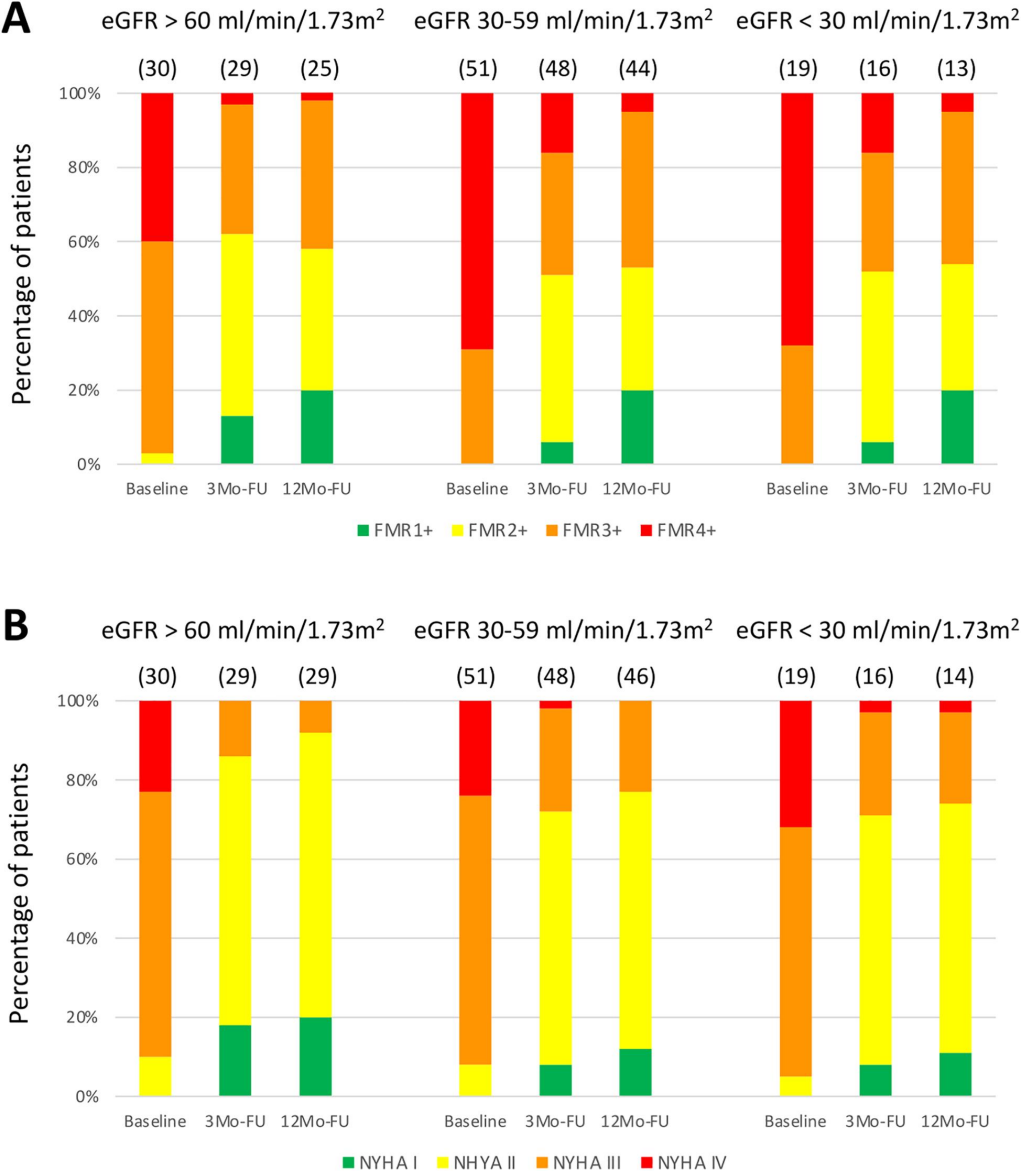
Clinical and echocardiographic assessment following indirect mitral annuloplasty in functional mitral regurgitation according to baseline renal function. Functional mitral regurgitation (FMR) class **(A)** and New York Heart Association (NYHA) classification **(B)** at baseline, 3-Months Follow-Up (3Mo-FU) and 12-Months Follow-Up (12Mo-FU). eGFR, estimated glomerular filtration rate.

FMR and NYHA classification following indirect mitral annuloplasty were improved at 3- and 12-months follow-up compared to baseline in all groups (Figure 4). Also, a linear mixed-effects model was applied to account for within-subject correlations. Fixed effects included timepoint, CKD groups (eGFR > 60 mL/min/1.73 m^2^, 30–59 mL/min/1.73 m^2^, <30 mL/min/1.73 m^2^), and their interaction. The analysis revealed a significant main effect of timepoint for NYHA (F[2,89.8] = 103.8, *p* < 0.001) and FMR severity (F[2,83.5] = 93.9, *p* < 0.001), indicating significant improvement over time in all groups. No significant main effects of renal function groups were observed for NYHA (F[2,92.0] = 1.29, *p* = 0.280) or FMR severity (F[2,90.6] = 1.31, *p* = 0.276). Additionally, no significant interaction effects between timepoint and renal function groups were found for NYHA (F[4,88.9] = 0.51, *p* = 0.731) or FMR severity (F[4,83.7] = 0.49, *p* = 0.741), indicating that the trajectories of improvement did not differ significantly between CKD groups. These results confirm that improvements in clinical status and mitral regurgitation severity over 12 months were consistent regardless of baseline renal function.

An interaction analysis was conducted to assess potential effect modification between AKI and the CKD groups. The interaction term between baseline renal function groups and AKI status on 1-year mortality was not statistically significant (*p* = 0.081), indicating no clear evidence for effect modification between the groups.

Following a multivariate analysis of baseline parameters, only baseline eGFR and vena contracta were independent predictors of 1-year mortality, whereas age, gender, diabetes, previous heart surgery, natural logarithm of NT-proBNP (ln NT-proBNP) and TAPSE failed to predict survival (Table 3). The univariate Cox regression analysis is displayed in Supplementary Table S1.

**Table 3:** Multivariate baseline predictors of 1-year mortality (Cox regression analysis).

Baseline parameters	Adjusted Hazard ratio (95% confidence interval)	*p*-Value
Age	0.94 (0.86–1.04)	0.231
Sex	0.48 (0.10–2.48)	0.383
Diabetes mellitus	0.72 (1.78–2.91)	0.645
Previous heart surgery	3.17 (0.69–14.66)	0.139
eGFR (mL/min/1.73 m^2^)	0.94 (0.90–0.98)	0.005
Vena contracta	1.70 (1.02–3.10)	0.044
TAPSE	1.01 (0.88–1.17)	0.864
Ln (NT-pro BNP)	0.99 (0.34–2.88)	0.989

eGFR, estimated glomerular filtration rate; TAPSE, tricuspid annular plane systolic excursion; Ln, natural logarithm.

## 4 Discussion

Indirect mitral valve annuloplasty using the Carillon Mitral Contour System has demonstrated efficacy in the treatment of FMR across multiple studies. The procedure is known for its favorable safety profile, with a low incidence of adverse events (1–3, 19). Our study verified a low rate of procedural complications, with no procedural deaths observed among a cohort of 100 patients.

Chronic kidney disease (CKD) is associated with a poor prognosis following mitral valve edge-to-edge repair (5, 19–22). In the setting of heart failure, kidney dysfunction is a recognized comorbidity, being considered a component of cardiorenal syndrome and is linked to decreased survival. Its impact on mortality may arise from venous congestion, forward heart failure, activation of the renin-angiotensin-aldosterone system (RAAS), and sympathetic stimulation associated with heart failure (5, 23, 24). To date, no clinical trial has investigated the impact of CKD in patients undergoing indirect mitral annuloplasty. Our study revealed the negative impact of CKD on survival following Carillon device implantation. Among the three eGFR groups, the cohort with CKD stage 4–5, defined as eGFR < 30 mL/min/1.73 m^2^, exhibited the poorest prognosis. This relationship remained consistent even after stratifying patients based on the occurrence of postprocedural AKI, with those experiencing AKI demonstrating the highest mortality rates. This finding is consistent with recent studies of FMR patients treated with mitral transcatheter edge-to-edge repair (TEER) (20–22).

In our cohort, patients who developed postprocedural AKI had higher baseline serum creatinine, lower eGFR, and a greater prevalence of coronary artery disease compared to those without AKI. Several mechanisms may contribute to the occurrence of AKI in this context. Pre-existing chronic kidney disease likely predisposes patients to impaired renal perfusion and increased vulnerability to hemodynamic changes during the procedure. In addition, periprocedural factors such as transient hypotension, venous congestion, or contrast medium exposure are well-known contributors to AKI, although these were not specifically assessed in our study. Finally, the higher burden of coronary artery disease observed in the AKI group may reflect a more advanced systemic atherosclerotic profile, which could further promote renal hypoperfusion and microvascular dysfunction.

The impact of indirect mitral annuloplasty in patients on chronic dialysis could not be assessed, as only one patient receiving dialysis at baseline was included. However, it may be hypothesized that outcomes in this population could be even less favorable compared to patients with CKD in the absence of dialysis. Further studies are needed to clarify this issue.

Numerous clinical trials have documented significant reductions in echocardiographic FMR parameters and favorable clinical outcomes, including improvements in echocardiographic measures such as EROA, PISA, regurgitant volume, and vena contracta in FMR patients undergoing indirect annuloplasty. Additionally, studies reported reductions in mitral annular dimensions and enhancement in NYHA functional class as well as the 6-Minute Walk Test performance (1–3, 25). Our findings further confirm these results, showing a reduction in quantitative MR parameters and NYHA class 12 months post-procedure following indirect mitral annuloplasty regardless of baseline renal function.

Consequently, while CKD stage 4–5 is associated with a worse prognosis, indirect annuloplasty improves quantitative MR parameters and subsequent MR-related symptoms. This finding might be explained by a reduction in left atrial pressure leading to cardiac decongestion.

Previous heart surgery and diabetes were associated with CKD, as patients with CKD typically present with a greater burden of comorbidities, including diabetes and advanced heart failure requiring cardiac surgery. Furthermore, reduced right ventricular function, indicated by decreased TAPSE, was more frequently observed in patients with CKD stage 4–5. However, pulmonary systolic artery pressure was similar in the three groups.

At baseline, NT-proBNP levels were significantly higher in group 3, as CKD is linked to more advanced heart failure and greater cardiac decongestion compared to a population of preserved renal function. A multivariate analysis confirmed baseline serum creatinine and eGFR as independent predictors of survival in our cohort. However, age, gender, diabetes, previous heart surgery, NT-proBNP and TAPSE were no predictors of mortality. These results underscore the importance of renal function regarding long-term survival in heart failure patients with FMR. Therefore, early optimal guideline directed medical therapy together with interventional FMR treatment might be needed for better preservation of renal function. Prevention of CKD is key in heart failure and early interventional strategies should be considered in local heart teams, while discussing patients with chronic kidney disease.

## 5 Conclusion

In summary, our study provides for the first time evidence that CKD as well as the presence of postprocedural acute kidney injury serve as significant predictors of impaired long-term survival in patients undergoing indirect mitral annuloplasty. However, indirect mitral annuloplasty enhances MR parameters and clinical symptoms over a 12-month follow-up period, even in the presence of impaired renal function. Consequently, Carillon device implantation seems to be a viable option for transcatheter treatment in FMR patients with or without CKD.

## 6 Limitations

Given the retrospective, single-center design and associated statistical limitations, the results of this study should be considered hypothesis-generating and warrant confirmation by prospective, randomized, multi-center trials. Furthermore, the relatively small cohort size (*n* = 100) limits the generalizability of the results and carries the risk of selection bias. Nevertheless, for indirect mitral annuloplasty using the Carillon device, our cohort represents one of the largest real-world series reported to date. Previous pivotal trials investigating the Carillon system (TITAN, *n* = 53; TITAN II, *n* = 36; and REDUCE FMR, *n* = 120) included comparable or smaller patient numbers. Moreover, patients with CKD in our study more frequently presented with comorbidities. Although we attempted to adjust for these imbalances using multivariate Cox regression analyses, residual confounding cannot be entirely excluded due to the observational nature of the study design. Improvements in NYHA classification and severity of FMR were observed irrespective of CKD. However, the lack of a control group in this retrospective study limits causal conclusions regarding the effect of the Carillon device. Changes in concurrent medical management or natural disease progression may also have contributed to the observed improvements. Therefore, these findings should be interpreted as associations rather than definitive proof of treatment effect. As this was a retrospective study, no chronicity criteria (e.g., ≥3 months of persistently reduced eGFR or albuminuria data) were directly available. During follow-up, renal parameters were not routinely assessed; therefore, changes in renal function potentially induced by successful Carillon device implantation could not be evaluated. Consequently, we were unable to investigate the trajectory of renal function after Carillon implantation, which limits conclusions about the potential impact of the procedure on subsequent renal function over time. While our data provide valuable hypothesis-generating insights, confirmation in larger, prospective, multicenter studies will be essential before these findings can be extrapolated to broader clinical practice.

## Data Availability

The raw data supporting the conclusions of this article will be made available by the authors, without undue reservation.
