# Supplementary material for: Chronic kidney disease and survival following indirect mitral annuloplasty for functional mitral regurgitation
Source: Front Cardiovasc Med. 2025 Dec 2;12:1622875. doi: 10.3389/fcvm.2025.1622875 (PMC12705570; doi:10.3389/fcvm.2025.1622875)
Supplement: Supplementary file 1 [file Table1.docx]

|  | **Carillon cohort**  **n = 100** | |  |  |
| --- | --- | --- | --- | --- |
|  | **n** | **% or Mean ± SEM** | **P-Value** | |
| Age | 100 | 80.5 ± 0.9 | 0.814 | |
| Male | 100 | 66.0 | 0.241 | |
| **Patients history** |  |  |  | |
| Arterial hypertension | 93 | 93.0 | 0.205 | |
| Diabetes mellitus | 26 | 26.0 | **0.067** | |
| Hyperlipidemia | 60 | 60.0 | 0.384 | |
| Smoker | 14 | 14.0 | 0.439 | |
| Coronary artery disease | 65 | 65.0 | 0.460 | |
| Previous heart surgery | 23 | 23.0 | **0.028** | |
| Atrial fibrillation | 78 | 78.0 | 0.652 | |
| **Transthoracic Echocardiography** |  |  |  | |
| LVEF (%) | 100 | 40.8 ± 1.3 | 0.205 | |
| LVEDD (mm) | 100 | 54.6 ± 0.8 | 0.203 | |
| Mitral annulus diameter (cm) | 100 | 4.3 ± 0.04 | 0.155 | |
| PASP (mmHg) | 100 | 45.6 ± 1.4 | 0.806 | |
| TAPSE (mm) | 100 | 17.8 ± 0.5 | **0.094** | |
| **FMR Grading** |  |  | 0.154 | |
| FMR 2+ | 1 | 1.0 | 0.638 | |
| FMR 3+ | 37 | 37.0 | 0.952 | |
| FMR 4+ | 62 | 62.0 | 0.895 | |
| Vena contracta (mm) | 100 | 5.9 ± 0.1 | **0.031** | |
| EROA (cm^2^) | 30 | 0.2 ± 0.02 | 0.460 | |
| PISA (mm) | 100 | 7.4 ± 0.1 | 0.437 | |
| Regurgitant Volume (ml) | 100 | 35.5 ± 1.6 | 0.755 | |
| **Dyspnea** |  |  | 0.217 | |
| NYHA II | 8 | 8.0 | 0.174 | |
| NYHA III | 67 | 67.0 | 0.798 | |
| NYHA IV | 25 | 25.0 | 0.372 | |
| **Laboratory Results** |  |  |  | |
| Ln (NT-proBNP) | 94 | 8.37 ± 0.8 | **0.015** | |
| Serum creatinine (mg/dl) | 100 | 1.5 ± 0.1 | **0.008** | |
| eGFR (ml/min/1.73m^2^) | 100 | 50.4 ± 1.9 | **0.003** | |
| Baseline dialysis | 1 | 1.0 | 0.638 | |
| Hemoglobin (g/dl) | 100 | 12.7 ± 0.2 | 0.724 | |
| **Baseline Medication** |  |  |  | |
| Beta blocker | 96 | 96.0 | 0.368 | |
| ACEI or ARB or ARNI | 89 | 89.0 | 0.812 | |
| MRA | 41 | 41.0 | 0.741 | |
| Diuretic | 97 | 97.0 | 0.412 | |

**Supplementary table 1. Univariate analysis of** **baseline predictors of 1-year mortality.** SEM=standard error of the mean; EF=ejection fraction; LV=left ventricle; LVEF=left ventricular ejection fraction; LVEDD=left ventricular enddiastolic diameter; PASP=systolic pulmonary artery pressure; TAPSE=tricuspid annular plane systolic excursion; FMR=functional mitral regurgitation; EROA=effective regurgitant orifice area; PISA=proximal isovelocity surface Area; NT-proBNP=N-terminal pro-B-type natriuretic peptide; NYHA=New York Heart Association; eGFR=estimated glomerular filtration rate; ACEI=angiotensin-converting enzyme inhibitor; ARB=angiotensin receptor blocker; ARNI=angiotensin receptor neprilysin inhibitor; MRA=mineralocorticoid receptor antagonist. Ln=natural logarithm.
